# Impact of the 2014 coinsurance rate revision for the elderly on healthcare resource utilization in Japan

**DOI:** 10.1186/s13561-021-00324-0

**Published:** 2021-07-06

**Authors:** Takumi Nishi, Toshiki Maeda, Susumu Katsuki, Akira Babazono

**Affiliations:** 1 Department of Research Planning and Information Management, Fukuoka Institute of Health and Environmental Sciences, 39 Mukaizano, Dazaifu-shi, Fukuoka, 818-0135 Japan; 2 Department of Public Health and Preventive Medicine, Fukuoka University, Fukuoka, Japan; 3 Department of Health Care Administration and Management, Graduate School of Medical Sciences, Kyushu University, Fukuoka, Japan

**Keywords:** Elderly, Health, Health insurance, Medical care, Public health

## Abstract

**Background:**

Cost sharing, including copayment and coinsurance, is often used to contain medical expenditure by decreasing unnecessary or excessive use of healthcare resources. Previous studies in Japan have reported the effects of a coinsurance rate reduction for healthcare from 30 to 10% on the demand for healthcare among 70–74-year-old individuals. However, the coinsurance rate for this age group has recently increased from 10 to 20%. This study aimed to estimate the economic impact of coinsurance rate revision on healthcare resource utilization.

**Methods:**

We collected claims data from beneficiaries of the municipality National Health Insurance and the Japanese Health Insurance Association in Fukuoka Prefecture. We categorized subjects born between March 2, 1944 and April 1, 1944 into the 20% coinsurance rate reduction group and those born between April 2, 1944 and May 1, 1944 into the 10% reduction group. An interrupted time-series analysis for multiple groups was employed to compare healthcare resource utilization trends before and after coinsurance rate reduction at 70 years.

**Results:**

The 10% coinsurance rate reduction led to a significant increase in healthcare expenditure for outpatient care. The 20% reduction group showed a significantly sharper increase in healthcare expenditure for outpatient care than the 10% reduction group. Similarly, the 10% coinsurance group significantly increased in the number of ambulatory visits. The 20% coinsurance rate reduction group had more frequent ambulatory care visits than the 10% reduction group.

**Conclusions:**

These results suggest that increasing the coinsurance rate among the elderly would reduce outpatient healthcare resource utilization; however, it would not necessarily reduce overall healthcare resource utilization.

## Background

Similar to other developed countries, Japan has a rapidly aging population. In 2020, 36.17 million people (28.7% of the total population) were aged 65 years or older [1]. One estimate suggests that the number and proportion of such older people will increase to 39.21 million (35.3%) in fiscal year (FY) 2040. Social security expenditure in Japan is expected to rapidly increase from approximately 121.3 trillion yen (21.5% of GDP) in FY 2018 to about 188.2–190.0 trillion yen (23.8–24.0% of GDP) in FY 2040 [2].

Higher patient cost sharing is often used to contain medical expenditure by reducing unnecessary or excessive use of healthcare resources. Using results from the famous RAND Health Insurance Experiment (RHIE) in 1974, Manning et al. examined cost-sharing effects on the demand for medical services and reported that price elasticity was approximately − 0.2. However, individuals aged 62 years or older at enrollment were intentionally excluded as ineligible groups in the RHIE [3]. Thereafter, Chandra et al. studied a policy change that raised patient cost sharing for supplemental insurers for retired public employees, including the elderly in California, and reported that elasticity for ambulatory care and prescription drugs was similar to that of RHIE [4]. However, these studies evaluated the effects of basic or supplemental insurance provision rather than patient cost sharing.

Patient cost sharing should be examined especially in rapidly aging countries, such as Japan, where among 43.07 trillion yen of national healthcare expenditure in FY 2017, 25.95 trillion yen (60.3%) was consumed by the elderly aged 65 years or older [5]. Nevertheless, little was known about the effects of cost sharing on healthcare utilization among the elderly, until few recent few studies in Japan. First, Shigeoka exploited a sharp reduction in patient cost sharing at age 70 in Japan by using patient survey data, death records, and the Comprehensive Survey of Living Conditions [6]. Fukushima et al. examined the effects of reducing the coinsurance rate from 30 to 10% for medical care on demand among Japanese people aged 70 years and older by analyzing claims data from health insurance societies [7]. These studies reported that both outpatient and inpatient care were price sensitive among the elderly. Further, considering the coinsurance rate revision for Japanese people aged 70–74 years, Mahlich and Sruamsiri evaluated the impact of increased coinsurance rates on drug, inpatient, and outpatient healthcare utilization among Japanese elderly with rheumatoid arthritis by analyzing administrative data from 147 acute care hospitals [8].

However, the overall situation remains unclear after the coinsurance rate revision for elderly people aged 70–74 years in 2014 for the following reasons. First, data analyzed by Shigeoka did not contain information about healthcare expenditure other than self-reported out-of-pocket medical spending [6]. Second, the study subjects of Fukushima et al. did not include beneficiaries of national health insurance (NHI), which is the most popular insurer for people aged 65–74 years in Japan [7]. Both evaluated the effects of reducing the coinsurance rate from 30 to 10% for medical care on demand among Japanese aged 70–74 years, but not after the coinsurance rate revision in 2014. Moreover, the study conducted by Mahlich and Sruamsiri did not include patients with medical conditions other than rheumatoid arthritis and medical institutions other than acute care hospitals [8].

Therefore, determining the effects of the coinsurance rate revision by comparison with the previous coinsurance rate would have important policy implications in healthcare economics. In this study, we estimated the economic impacts of the coinsurance rate revision on healthcare resource utilization by analyzing longitudinal health insurance claims data. Further, although previous studies implemented age-based regression discontinuity design, we employed interrupted time-series analysis (ITSA), which is a useful quasi-experimental design for evaluating the longitudinal effects of the coinsurance rate revision. As a result, we found that the coinsurance reduction from 30 to 20% also increased the utilization of outpatient healthcare resources, although its impact was smaller than with a 20% reduction.

### Institutional setting

In Japan, since universal health coverage was achieved in 1961, almost every person is covered by public health insurance. Although several types of insurers are available in Japan, such as NHI, Japanese health insurance association (JHIA), health insurance societies, mutual aid associations, and medical care systems for the elderly in the later stages of life, medical service fees are reimbursed based on a nationally uniform fee schedule. Many outpatient and inpatient services were reimbursed by fees for service schemes; however, in 2003, a prospective payment system for acute inpatient services was introduced. The diagnosis procedure combination per diem payment system (DPC/PDPS) is a prospective payment system applied on a per diem basis and covers about 54% of general hospital beds. The prices of prescription drugs and specific medical devices were also regulated nationally through uniform price lists.

Beneficiaries have free access to each type of medical institution authorized for providing medical, dental, and pharmaceutical services covered by health insurance. However, additional copayment is required for the first visit to a large hospital without a physician’s referral. Moreover, other than catastrophic coverage, patient bill copayment at medical institutions and the rest of the fees would be reimbursed by insurers.

### Cost-sharing reforms in Japan

The Japanese government implemented several cost-sharing reforms. First, copayments (inpatients, 300 yen/day; outpatients, 400 yen/day) for the elderly aged 65 years or older were introduced in February 1983; however, these individuals did not have to pay out-of-pocket expenses from January 1973 until February 1983. Subsequently, with the reform of Japan’s health insurance system in January 2001, 10% coinsurance was introduced. Then, coinsurance rate for the elderly with high income which is comparable to the current workforce was increased to 20% in October 2002, and it was increased to 30% in October 2006. Further, owing to a rapid increase in medical expenditure for older people, in 2008, the government implemented an independent health insurance system for individuals aged 75 years or older as part of the medical care system for the elderly in the latter stage of life. Accordingly, the coinsurance rate for people in Japan aged 70–74 years increased from 10 to 20%, after the amendment of the Health Insurance Act and National Health Insurance Act [9, 10]. To mitigate the impact of rapid change, the coinsurance rate was frozen at 10% except by budgetary provision after 2008. However, the 20% coinsurance rate was enforced for people who reached 70 years of age after April 2014 to reduce inequity across generations. Therefore, since FY2014, the coinsurance rate for people who reached 70 years of age (i.e., those who were born after April 2, 1944) decreased from 30% (for people aged 6–69 years of age) to 20% (for people aged 70–74 years of age) after their birth month, while the coinsurance rate for people who had already reached 70 years of age (i.e., those who were born before April 1, 1944) decreased from 30 to 10%.

### Data

**Fig. 1:**
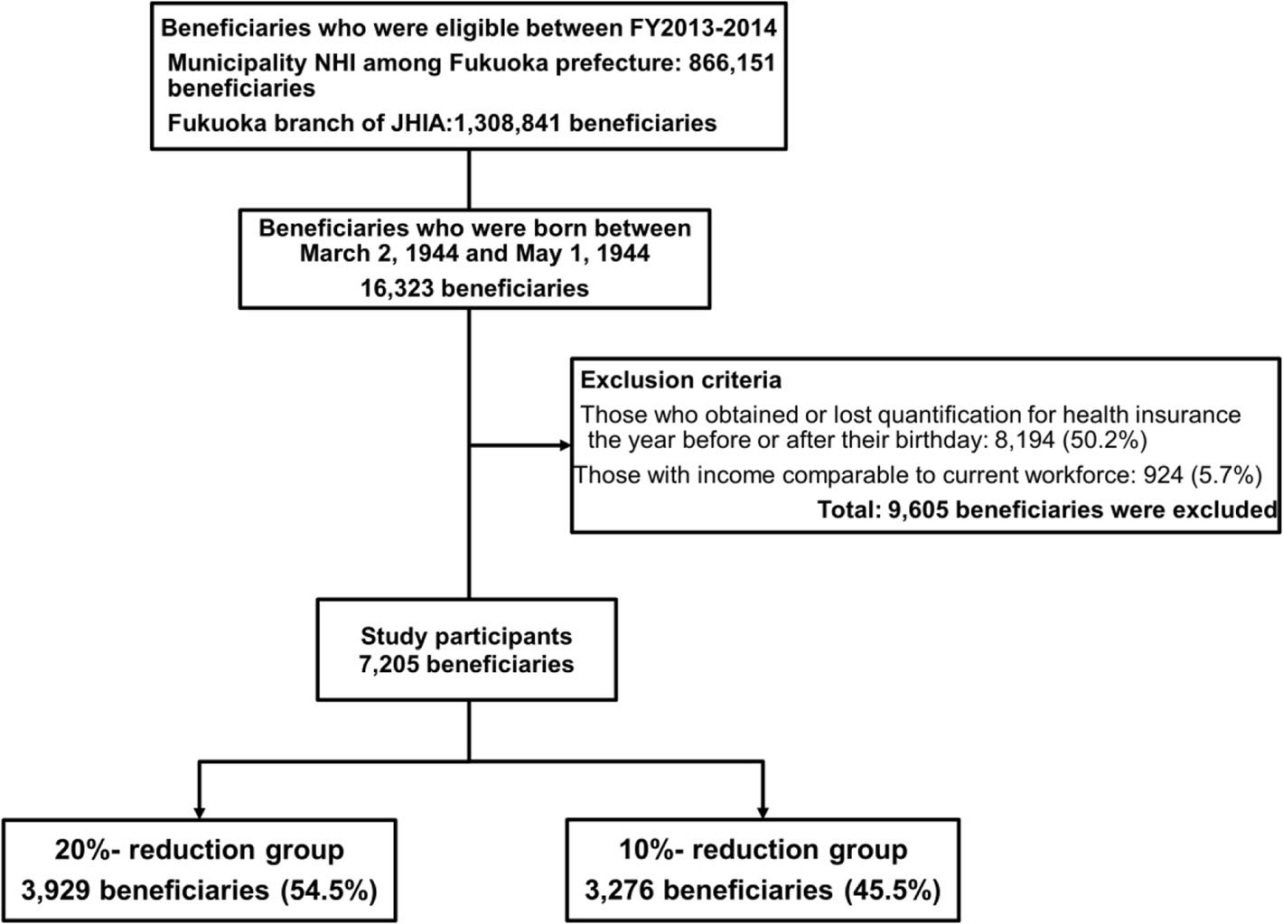
Flowchart of inclusion and exclusion criteria

The data of health insurance beneficiaries aged 70 years in FY 2013 or 2014 and their health insurance claims data other than that of dental care were obtained from the claims database of municipality NHI in Fukuoka Prefecture and the Fukuoka branch of the JHIA. We collected claims data before and after 12 months from the next month of the 70th birthdays of 16,353 beneficiaries who were born between March 2, 1944 and May 1, 1944. Figure 1 presents the inclusion and exclusion criteria and the subject selection flow chart. First, we excluded 8194 individuals who obtained or lost quantification for health insurance the year before or after their birthday. Further, we excluded 955 beneficiaries with income comparable to the current workforce, that is, those who did not change their coinsurance rates. Finally, 7205 beneficiaries were selected as study subjects. We categorized subjects born between March 2, 1944 and April 1, 1944 and whose coinsurance rates were to decrease to 10% the following month upon reaching 70 years of age into the 20% (coinsurance rates) reduction group. We classified those born between April 2, 1944 and May 1, 1944 and those whose insurance rates decreased to 20% into the 10% reduction group.

Moreover, health insurance beneficiaries can receive catastrophic coverage, as shown in Table 1. As catastrophic coverage was delivered in kind for beneficiaries who presented the “Eligibility Certificate for Ceiling-Amount Application” or an “Eligibility Certificate for Ceiling-Amount Application and Reduction of the Standard Amount of Patient Liability,” we computed actual out-of-pocket payments by using information on out-of-pocket payments for catastrophic coverage. Subsequently, we calculated the actual coinsurance rate, dividing this by total healthcare expenditure. The actual coinsurance rates of the 10% reduction group before and after 70 years of age were 23.5 and 13.1% in overall settings, 29.5 and 18.0% for outpatient care, and 11.7 and 6.5% for inpatient care. We used the 2010 exchange rate (US$1 = 87.75 yen) for easy comparison with the study results reported by Fukushima et al. [7].

**Table 1:** The amounts of catastrophic coverage

	Average annual income	**Catastrophic coverage: maximum out-of-pocket expenditure per month (yen)**	
**Aged under 70 years**	Over approximately 11,600,000 yen	252,600 + (medical expenditure – 842,000) × 1%	
	About 7,700,000 to 11,600,000 yen	167,400 + (medical expenditure – 558,000) ×1%	
	About 3,700,000 to 7,700,000 yen	80,100 + (medical expenditure – 267,000) × 1%	
	Under approximately 3,760,000 yen	57,600	
	Exempted from residence tax	35,400	
	Income levels	Outpatient	Inpatient
**Aged 70 to 74 years**	High income (Comparable to current workforce)	44,400	80,100 + (medical expenditure −267,000) × 1%
	Regular income	12,000	44,400
	Low income II (Exempt from residence tax)	8000	24,600
	Low income I (Particularly low income)		15,000

We employed medical expenditure and the number of outpatient visits or the length of inpatient stay as the main outcome variables. These variables were also separately measured according to inpatient and outpatient settings.

Descriptive statistics of the study subjects are shown in Table 2. The gender proportions and types of health insurance were similar between the two groups. The 20% reduction group had higher healthcare expenditure during the 2-year study period (approximately $963.2) than the 10% group. Similarly, a difference in the number of outpatient visits was observed (approximately 3.2 days).

**Table 2:** Descriptive statistics of study subjects

	Coinsurance rates change			
	*20% reduction*		*10% reduction*	
	(*N* = 3929)		(*N* = 3276)	
*Gender, N (%)*				
Male	1779	(45.3%)	1420	(43.3%)
Female	2150	(54.7%)	1856	(56.7%)
*Type of health insurance, N (%)*				
JHIA	454	(11.6%)	407	(12.4%)
NHI	3475	(88.4%)	2869	(87.6%)
**Healthcare resource utilization in all settings**				
*Healthcare expenditure, mean (sd)*				
Before reaching 70 years old	4255.0	(9168.0)	3664.4	(7178.2)
After reaching 70 years old	5225.9	(10,388.0)	4853.2	(9751.7)
Overall	9480.9	(16,631.8)	8517.7	(14,773.5)
Healthcare expenditure > 0	3669	(93.4%)	3050	(93.1%)
*Treatment days (No. of ambulatory visit/ length of inpatient stay), mean (sd)*				
Before reaching 70 years old	23.4	(36.8)	22.3	(35.9)
After reaching 70 years old	28.8	(41.8)	26.3	(40.1)
Overall	52.2	(73.7)	48.6	(72.1)
**Healthcare resource utilization for outpatient care**				
*Healthcare expenditure, mean (sd)*				
Before reaching 70 years old	2448.6	(3116.3)	2426.6	(3220.2)
After reaching 70 years old	3018.4	(3590.0)	2770.7	(3663.1)
Overall	5467.0	(6257.2)	5197.3	(6590.4)
Outpatient expenditure > 0	3661	(93.2%)	3036	(92.7%)
*No. of ambulatory visit, mean (sd)*				
Before reaching 70 years old	19.0	(24.6)	18.4	(22.1)
After reaching 70 years old	23.3	(28.7)	20.8	(24.0)
Overall	42.4	(49.6)	39.2	(43.1)
**Healthcare resource utilization for inpatient care**				
*Healthcare expenditure, mean (sd)*				
Before reaching 70 years old	1806.4	(8180.6)	1237.9	(6113.4)
After reaching 70 years old	2207.5	(9252.3)	2082.5	(8623.4)
Overall	4014	(14,381.7)	3320	(12,487.0)
Inpatient expenditure > 0	860	(21.9%)	664	(20.3%)
*No. of admission, mean(sd)*				
Before reaching to 70 years old	0.2	(0.5)	0.1	(0.4)
After reaching to 70 years old	0.2	(0.7)	0.2	(0.6)
Overall	0.4	(1.0)	0.3	(0.8)
*Length of inpatient stay, mean (sd)*				
Before reaching 70 years old	4.4	(26.8)	3.9	(28.9)
After reaching 70 years old	9.8	(53.6)	9.4	(59.0)
Overall	5.5	(30.3)	5.5	(32.6)

*NHI* National Health Insurance, *JHIA* Japanese Health Insurance Association

## Methods

**Fig. 2:**
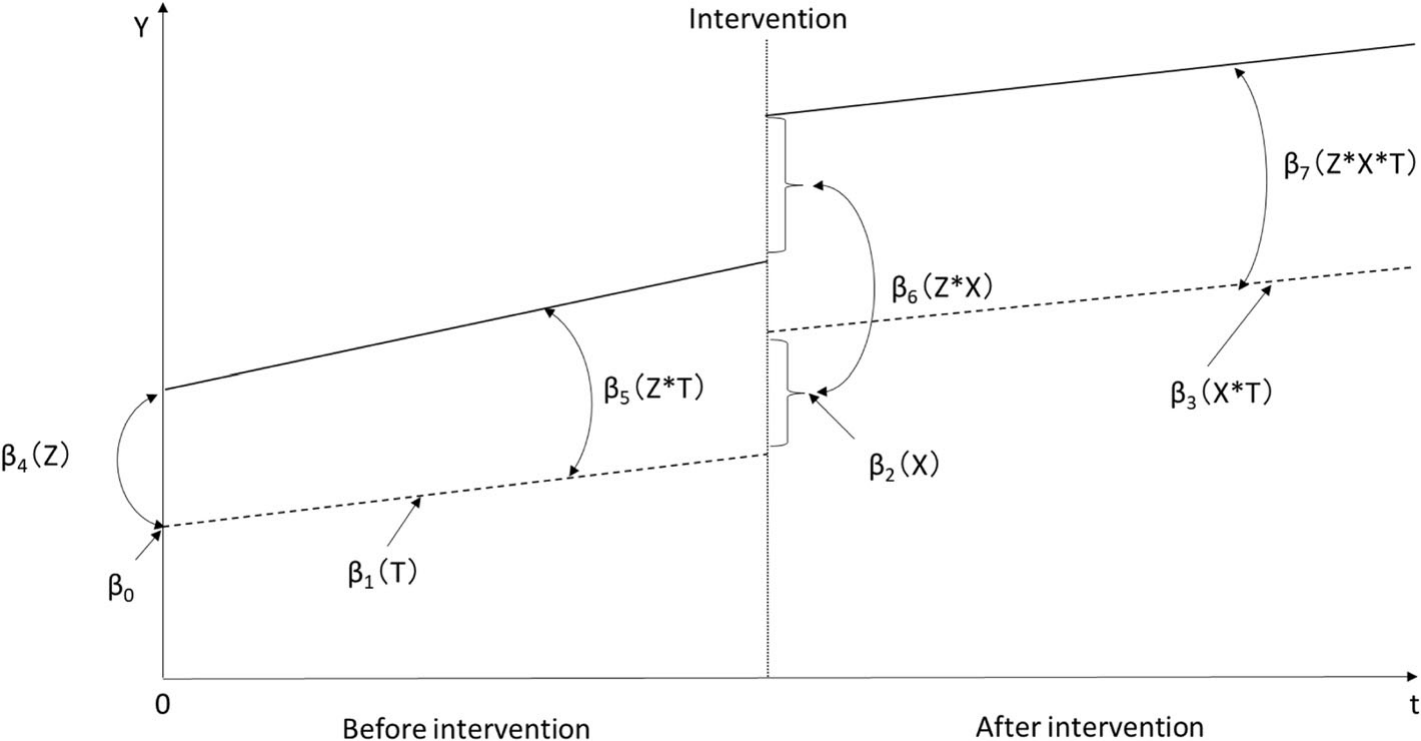
Visual depiction of multiple group ITSA

We assigned the 10% reduction group as the control group (reference) and the 20% reduction group as the assigned treatment group. Thereafter, as conventional regression discontinuity design could not estimate the difference in multiple groups, the ITSA for multiple groups was employed to compare healthcare resource utilization trends before and after intervention (coinsurance rate reduction at 70 years). The ITSA offers a quasi-experimental research design for observational studies [11]. The visual depiction of multiple group ITSA is presented in Fig. 2. The solid line indicates the treatment group, and the dotted line shows the control group. The ITSA model used the following equation [12]:

$$ {Y}_{\mathrm{t}}={\beta}_0+{\beta}_1{T}_t+{\beta}_2{X}_t+{\beta}_3{X}_t{T}_t+{\beta}_4\mathrm{Z}+{\beta}_5{ZT}_t+{\beta}_6{ZX}_t+{\beta}_7Z{X}_t{T}_t+{\upvarepsilon}_t $$

where Y_t_ is the outcome measure along time t; T_t_ is a time variable based on the point when the study began; X_t_ is a dummy variable indicated as 0 before and 1 after intervention; Z is a dummy variable for assignment to 0, the control, or 1, the treatment group. β_0_ to β_3_ represent trends in the control group as follows: β_0_, intercept; β_1_, slope before the intervention; β_2_, change in the trend caused by the intervention; β_3_, coefficient of the interaction between X_t_ and T_t_, and the slope after the intervention. β_4_ to β_7_ represent differences between control and treatment groups as follows: β_4_, difference in the intercepts; b_5_, difference in the slopes before the intervention; β_6_, difference in the changes caused by the intervention; β_7_, difference in the slopes after the intervention [12]. After individual data were organized, we generated the ITSA dataset by aggregating individual data by month. And we employed Prais regression (Prais–Winsten estimation), as statistical analyses used for ITSA must account for autocorrelated data [13]. All statistical analyses were performed using Stata 15.1 for Windows (Stata Corp, College Station, TX, USA). We then used the ITSA command [13].

## Results

### Total healthcare resource utilization

**Fig. 3:**
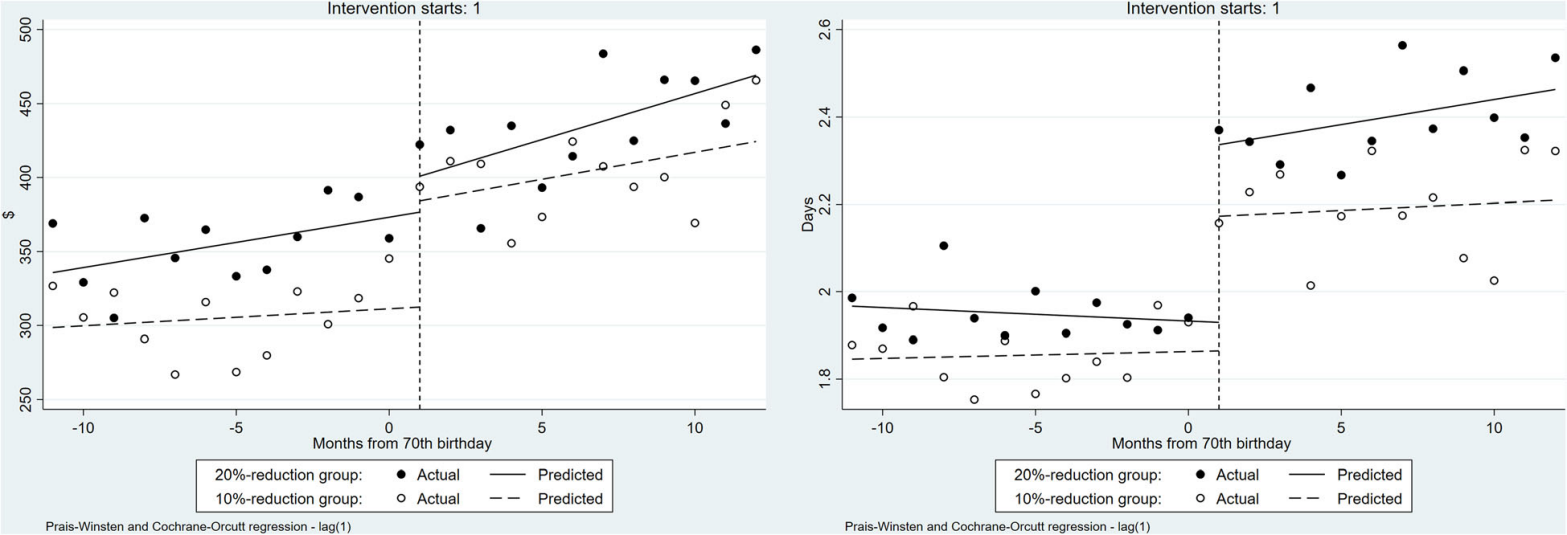
Healthcare resource utilization before and after the month of reaching 70 years of age (US dollars, days)

Table 3 presents the results of the total healthcare resource utilization obtained with the ITSA. Figure 3 presents the observed and predicted values according to the treatment setting. The solid lines and filled circles indicate the treatment group (20% reduction group), while the dotted lines and open circles show the control group: 10% reduction group.

**Table 3:** Results for healthcare resource utilization by ITSA

		Coeff.			
		(Std.err)			
		Expenditures		Treatment days	
β_0_:	Intercept	298.60	***	1.85	***
		(12.20)		(0.04)	
β_1_:	Trend before the 10% coinsurance rate reduction	1.15		0.00	
		(1.93)		(0.01)	
β_2_:	Change in the trend caused by 10% coinsurance rate reduction	71.73	**	0.31	***
		(20.58)		(0.07)	
β_3_:	Trend after the 10% coinsurance rate reduction	2.50		0.00	
		(3.21)		(0.01)	
β_4_:	Difference in the intercepts	37.21	**	0.12	**
		(18.35)		(0.05)	
β_5_:	Difference in the slopes before the coinsurance rate reductions	2.26		0.00	
		(2.72)		(0.01)	
β_6_:	Difference in the changes in the trend caused by coinsurance rate reductions	−47.65		0.10	
		(28.51)		(0.08)	
β_7_:	Difference in the slopes after the coinsurance rate reductions	0.31		0.01	
		(4.21)		(0.01)	
	R^2^	0.85		0.92	
	Observations	48		48	

^***^p < 0.001, ^**^p < 0.05, ^*^p < 0.1

The 10% coinsurance rate reduction led to a significant increase in healthcare expenditure: the coefficient (β_2_) representing the change in the trend caused by the coinsurance rate reduction in the 10% reduction group was 71.73 (*P* = 0.001). And there was a significant increase in treatment days: β_2_ was 0.31 (*P* < 0.001). However, the coefficient (β_6_) representing the difference in the changes in the trend among coinsurance rate reductions was − 47.65 (*P* = 0.102) in healthcare expenditure and treatment days (β_6_) was 0.10 (*P* = 0.200).

### Healthcare resource utilization for outpatient care

**Fig. 4:**
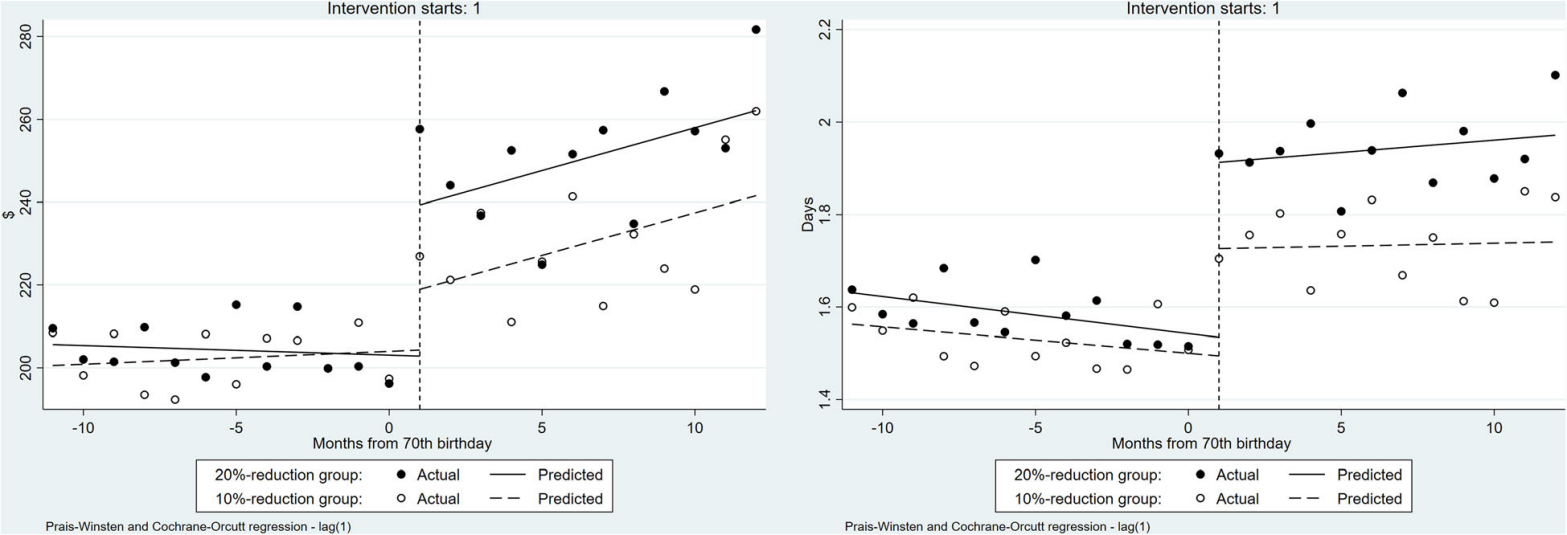
Healthcare resource utilization for outpatient care before and after the month of reaching 70 years of age

Table 4 and Fig. 4 show the results of healthcare resource utilization for outpatient care obtained with the ITSA. The 10% coinsurance rate reduction led to a significant increase in healthcare expenditure for outpatient care: β_2_ was 14.62 (*P* = 0.018). The 20% reduction group showed a significantly sharper increase in healthcare expenditure for outpatient care than the 10% reduction group; β_6_ was 21.87 (*P* = 0.013). Similarly, the 10% coinsurance group significantly increased in the number of ambulatory visits: β_2_ was 0.23 (*P* < 0.001). The 20% coinsurance rate reduction group had more frequent ambulatory care visits than the 10% reduction group: β_6_ was 0.15 (*P* = 0.019).

**Table 4:** Results for healthcare resource utilization for outpatient care by ITSA

		Coeff.			
		(Std.err)			
		Expenditures		No. of ambulatory visit	
β_0_:	Intercept	200.56	***	1.56	***
		(2.80)		(0.02)	
β_1_:	Trend before the 10% coinsurance rates reduction	0.31		−0.01	
		(0.38)		(0.00)	
β_2_:	Change in the trend caused by 10% coinsurance rates reduction	14.62	**	0.23	***
		(5.91)		(0.05)	
β_3_:	Trend after the 10% coinsurance rates reduction	1.74		0.01	
		(1.16)		(0.01)	
β_4_:	Difference in the intercepts	5.07		0.07	**
		(3.47)		(0.03)	
β_5_:	Difference in the slopes before the coinsurance rate reductions	−0.54		0.00	
		(0.54)		(0.01)	
β_6_:	Difference in the changes in the trend caused by coinsurance rate reductions	21.87	**	0.15	**
		(8.46)		(0.06)	
β_7_:	Difference in the slopes after the coinsurance rate reductions	0.56		0.01	
		(1.51)		(0.01)	
	R^2^	0.92		0.91	
	Observations	48		48	

^***^p < 0.001, ^**^p < 0.05, ^*^p < 0.1

### Healthcare resource utilization for inpatients care

**Fig. 5:**
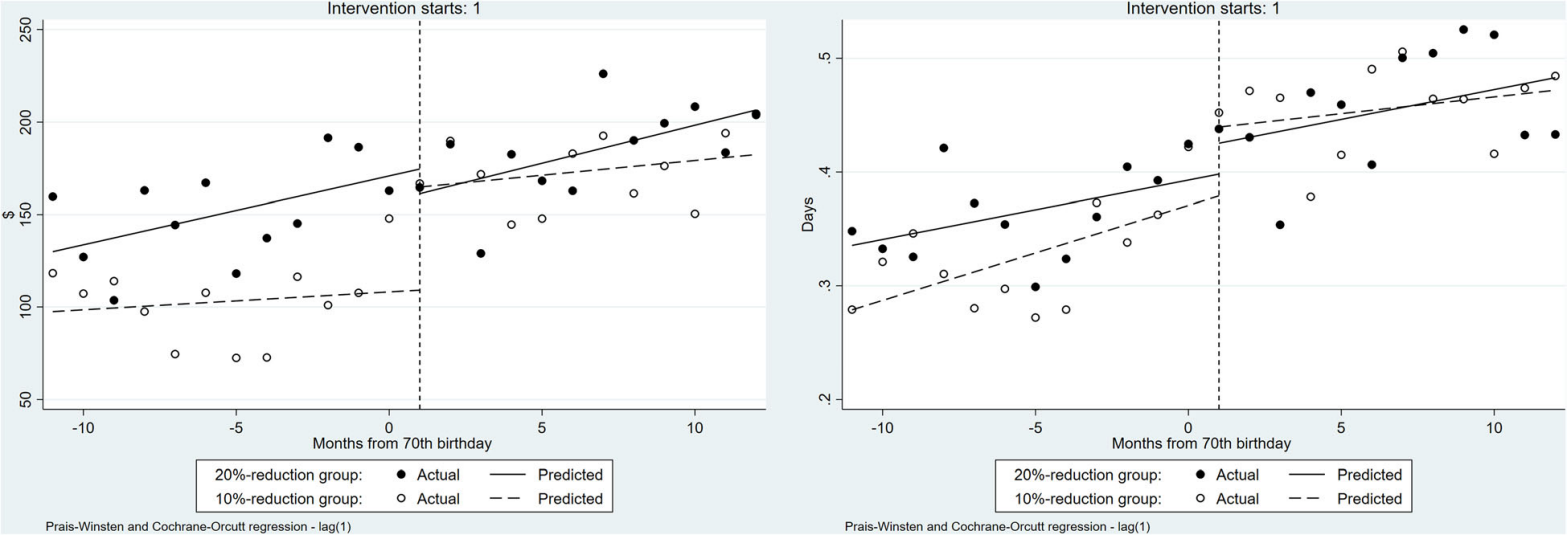
Healthcare resource utilization for inpatient care before and after the month of reaching 70 years of age

Table 5 and Fig. 5 show the results of healthcare resource utilization for inpatient care obtained with the ITSA. The 10% coinsurance rate reduction led to an increase in inpatient healthcare resource utilization: β_2_ was 55.72 for expenditure (*P* = 0.005) and 0.06 (*P* = 0.065) for length of inpatient stay. In addition, the 20% reduction group tended to have fewer changes in inpatient expenditure than the 10% reduction group: β_6_ was − 69.05 (*P* = 0.011), while no significant differences was found in the length of inpatient stay among coinsurance rate reduction groups.

**Table 5:** Results for healthcare resource utilization for inpatient care by ITSA

		Coeff.			
		(Std.err)			
		Expenditures		Length of inpatient stay	
β_0_:	Intercept	97.48	***	0.28	***
		(10.28)		(0.02)	
β_1_:	Trend before the 10% coinsurance rates reduction	0.97		0.01	**
		(1.92)		(0.00)	
β_2_:	Change in the trend caused by 10% coinsurance rates reduction	55.72	**	0.06	*
		(18.84)		(0.03)	
β_3_:	Trend after the 10% coinsurance rates reduction	0.63		−0.01	
		(2.44)		(0.00)	
β_4_:	Difference in the intercepts	32.50	*	0.06	**
		(16.44)		(0.03)	
β_5_:	Difference in the slopes before the coinsurance rate reductions	2.75		0.00	
		(2.65)		(0.00)	
β_6_:	Difference in the changes in the trend caused by coinsurance rate reductions	−69.05	**	−0.03	
		(25.71)		(0.04)	
β_7_:	Difference in the slopes after the coinsurance rate reductions	−0.22		0.01	
		(3.36)		(0.01)	
	R^2^	0.76		0.59	
	Observations	48		48	

^***^p < 0.001, ^**^p < 0.05, ^*^p < 0.1

## Discussion

We found that a 10% coinsurance rate reduction also tended to increase the utilization of outpatient healthcare resources. When comparing 10% coinsurance rate reduction with that of 20%, the increases in health care resource utilization was larger for the 20% reduction group than the 10% reduction group, particularly in the outpatient setting. Unlike previous investigations, the present study evaluated not only the effects of decreased coinsurance at age 70 on healthcare resource utilization under the revised coinsurance rate but also examined differences in the period before and after coinsurance rate revision. Substantial increases and differences among coinsurance rate groups were evident only in outpatient healthcare resource utilization immediately after the age of 70. These results were consistent with those of a previous study [7]. However, Shigeoka reported a clear effect of the reduction in inpatient admission after individuals were 70 years old [6]. These inconsistent results could be because of the difference in study design and data whether longitudinal claims data or repeated cross-sectional survey data were used. Moreover, conditions demanding inpatient care are severe or critical, and although we removed beneficiaries who died during the study period, some results for inpatient healthcare resource utilization were unclear. This is especially true in more expensive inpatient care, as the municipality NHI was the insurer for most of the people aged 65–74 years, this study’s subjects would be less healthy than that of the previous study. Indeed, compared with the probability of Fukushima et al., which was 0.652 and 0.0187 per person-month for outpatient and inpatient care, respectively, the proportion of our study subjects who visited ambulatory care at least once during the study period was 92.9% and that of those hospitalized was 21.2%. Additionally, the higher coinsurance rates are, the more applicable the catastrophic coverage becomes, because the 10% reduction group tended to reach the threshold of healthcare expenditure and the effect of coinsurance rate revision could be offset by the effect of out-of-pocket caps in expensive treatment. (in outpatients care; 10% coinsurance rate: 120,000 yen vs 20% coinsurance rate: 60,000 yen, in inpatient care; 444,000 yen vs 222,000 yen). Therefore, as we did not exclude the participants who received catastrophic coverage, the counteracted effect could nave led to unclear result related to inpatient healthcare resource utilization which tended to be more expensive than outpatient care.

Further, patients would postpone outpatient care, which would be deferred, as they knew that their coinsurance rates decreased at 70 years of age. Recently, Lin and Sacks reported that subjects in deductible plans increased spending and utilization to hit the maximum dollar expenditure in the last 3 months of every coverage year, and those in free care also increased at the end of the experiment year, except for acute care, by analyzing the data from the RAND Health Insurance Experiment [14]. Their results provided strong evidence of intertemporal substitution. Thus, our results imply that outpatient healthcare resource before and after 70 years of age would be affected by intertemporal substitution.

However, this study has several limitations. First, the sample size of this study was smaller than that of previous reports. Thus, sub-analyses, such as stratified analyses by clinical departments, could not be performed. Second, as observation periods in this study were shorter than those in previous investigations, our study subjects did not tend to hospitalize. Third, there may still remain selection bias because our data included beneficiaries only in a Fukuoka prefecture. Moreover, our data did not include beneficiaries of health insurance societies and mutual-aid associations. The total population aged 70–74 years in Fukuoka prefecture was 292,231 on April 1st, 2015 [15], and the number of beneficiaries older than 70 years were 216,243 in municipality NHI in Fukuoka prefecture and 15,714 in the Fukuoka branch of JHIA at the end of FY 2014 [16, 17]. Thus, our data covered at least 79.3% of the total population, although we could not collect information about the number of older adults receiving public assistance who were not covered by health insurance. Therefore, further research should be conducted to reveal the long-term effects of coinsurance rates revision. Additionally, public and political concerns about coinsurance rate revision for those older than 75 years has recently been growing; however, insurers for this age group are independent of other insurers. In this study, we developed a longitudinal database including long-term care insurance claims and health check-ups data, enabling us to investigate the long-term effect of the coinsurance rate revision throughout various insurers. Thus, we could examine the effects of coinsurance rate reduction for the 10% reduction group at 75 years on health status and long-term care resource utilization in the near future, after the beneficiaries who were born after April 2, 1944 reached 75 years of age in FY2019.

## Conclusion

In this study, we estimated the economic impacts of the 2014 coinsurance rate revision on healthcare resource utilization in Japan. We analyzed longitudinal health insurance claims data of municipality NHI in Fukuoka Prefecture and the Fukuoka branch of the JHIA by using ITSA. As a result, we found that the coinsurance reduction from 30 to 20% also had an impact on the outpatient healthcare resources utilization. Further, we observed that the impact was smaller than with a 20% reduction in outpatient care resource utilization, while it was larger for inpatient healthcare expenditure.

Our results provide the new finding that substantial increases and differences caused by coinsurance rate revision were evident only in outpatient healthcare resource utilization. Further, our findings contribute to the policy debates for cost sharing by elderly people in Japan. In conclusion, we clarified that increasing the coinsurance rate among the elderly in Japan would reduce outpatient healthcare resource utilization; however, it would not necessarily reduce overall healthcare resource utilization.

## Data Availability

We cannot make our data publicly available because the insurers did not provide permission to do so.

## Abbreviations

FY: Fiscal year
NHI: National health insurance
ITSA: Interrupted time-series analysis
JHIA: Japanese health insurance association
DPC/PDPS: Diagnosis procedure combination per diem payment system
